# Supplementary material for: Iterative improvement in the automatic modular design of robot swarms
Source: PeerJ Comput Sci. 2020 Dec 7;6:e322. doi: 10.7717/peerj-cs.322 (PMC7924708; doi:10.7717/peerj-cs.322)
Supplement: Supplemental Information 3 [file peerj-cs-06-322-s003.zip › argos3/doc/api_embedded_header.php]

php include "php/utility.php"; ?


The ARGoS Website
$treeview
$search
$mathjax


ARGoS  
Large-scale robot simulations

- home
- about
- download
- documentation

  

- examples
- user manual
- dev manual
- API
- forum
- publications


$searchbox
